# Are academic conferences serving their purpose? A survey among faculties and delegates of a national level orthopedic conference in a developing country

**DOI:** 10.11604/pamj.2023.44.4.37404

**Published:** 2023-01-04

**Authors:** Arghya Kundu Choudhury, Aman Verma, Nikhil Goyal, Tarun Goyal, Pankaj Kandwal, Shobha Arora, Sitanshu Barik

**Affiliations:** 1 Department of Orthopedics, All India Institute of Medical Sciences, Rishikesh, India,; 2 Department of Orthopedics, All India Institute of Medical Sciences, Bathinda, India,; 3 Department of Orthopedics, All India Institute of Medical Sciences, Deoghar, India

**Keywords:** Academic utility, conference, medical education, professional socialization, survey

## To the editors of the Pan African Medical Journal

There is a lack of enough evidence in determining the actual usefulness of conferences and continuing medical education (CME) programs among clinicians with active practice [1,2]. This survey aimed at studying the various motives behind attending orthopedic conferences and analyzing the attitude, perceptions, and practices towards these activities among the faculties and the delegates. A cross-sectional questionnaire-based study was conducted among all delegates and faculties, attending the annual conference of national-level orthopaedic conference from 14^th^-16^th^ February 2020, at a university-level teaching hospital in a developing country. The focus of the questionnaire was the following: expectations and motivations for conference participation, the importance of conferences for the scientific qualification process, interaction, and communication among colleagues, and individual pay-off of the conference visit. A pilot study was conducted including 10 orthopaedic surgeons for testing comprehensibility, ease of reading, and acceptability of questionnaires. The questionnaires were kept confidential and collected with strict anonymity from all the participants. A total of 197 completed response sheets, were considered for final analysis. Out of the total participants, 29.9% (n=59) were faculties and 70.1% (n=138) were delegates. Forty-four point two percent (44.2%; n=61) delegates and 71.2% (n=42) faculties were frequent conference-goers.

According to the delegates, the main motivation behind attending scientific conferences was to present their work. Sixty point one percent (60.1%; n=83) strongly agreed, and another 23.2% (n=32) agreed to this response. (n=83) strongly agreed, and another 23.2% (n=32) agreed to this response. Seventy-four point seven (74.7%; n=103) of delegates strongly agreed/agreed that they were directed by their teaching faculty to attend the conference. The majority of the delegates were unsure whether attending conferences was added to their curriculum vitae (CV) (61.6%, n=85). The academic content was the most important factor according to 89.1% (n=123) delegates. Seventy-one percent (71%; n=98) of delegates strongly agreed/agreed that venue and location were important for the conference. Conference kits, quality of the food and beverages, and cultural programs were strongly agreed/agreed upon as important by only 14.5%, 74.7%, and 76.1% of participants respectively. Interestingly, 60.1% (n=83) of delegates reported that presenting a paper was more important than publishing their research. Overall, 54.3% (n=75) of delegates agreed that conferences are truly academic events.

According to the faculties, the primary motivation behind attending conferences was also to present their research work with 55.9% (n=33) strongly agreeing while 20.3% (n=12) agreeing with this response. Adding to their CV for future endeavors was strongly agreed/agreed upon by 59.3% (n=35) faculty. Only 37.3% (n=14) strongly agreed/ agreed that they attend conferences to oblige their friends while a majority 45.8% (n=27) remained neutral to the response. Eighty-six point four percent (86.4%; n=51) and 89.7% (n=53) strongly agreed/ agreed that they attend a conference to make professional connections and to visit the local place respectively. The majority of them were motivated by the talks given by other faculties, and most of them picked up a research topic from the earlier conferences attended by them. Most of them agreed that they have changed their teaching/clinical practice from the knowledge gained from previous conferences. According to 52.5% (n=31) of the attending faculties, the conference is more of an academic event, while 61% (n=36) agreed that publishing a scientific work remains more important than only presenting it at these meetings.

Comparing the responses of both delegates and faculties, the main motivation behind their attendance was, identified to present their research work (p= 0.529) and the quality of the conference was found to depend mainly on academic content (p=0.203). Responses of frequent conference-going delegates and faculties were also compared and all questions had similar responses except, most of them did not attend the full conference. Publishing research work was significantly noted to be more important (p=0.006) among the faculties than only presenting the research work, at a scientific conference. The strengths of the study are the anonymity of responses, two different well-structured questionnaires catering to the perceptions of both delegates and faculties, and a national-level medical conference. Further studies with a larger number of participants for validating this questionnaire-based survey may be considered in the future. An online survey engaging a larger number of participants would have been better for an overall analysis of the actual academic utility of scientific conferences.

## Conclusion

These conferences help in developing interest in research works and in picking up topics for future research activities. Previous studies too highlight that the attending faculties and delegates were greatly motivated by the talks and presentations given by other faculties and it helped them to update their knowledge for improving the healthcare delivery system [3-5]. To some extent, present times have witnessed the grandeur and glitz of a scientific conference are mainly dependent on the increasing commercialization and marketing, with an interest to visit newer places [6,7]. Judicious use of resources, more so in developing countries, is needed to fully imbibe the benefits as well as attractions of conferences.
